# Electrochemical skin conductance: a tool for risk stratification and early anticipation of diabetic foot ulcers

**DOI:** 10.3389/fendo.2025.1437858

**Published:** 2025-03-17

**Authors:** Jean-François Gautier, Jean-Pierre Riveline, Louis Potier, Olivier Bourron, Lyse Bordier, Benjamin Vittrant, Ronan Roussel, Bernard Bauduceau

**Affiliations:** ^1^ Diabetology and Endocrinology Department, Lariboisière Hospital, Paris, France; ^2^ Institut Necker-Enfants Malades, Université Paris Cité, Institut National de la Santé et de la Recherche Médicale (INSERM) UMR-S1151, CNRS, Paris, France; ^3^ Diabetology – Endocrinology Department, Bichat-Claude-Bernard Hospital, Assistance Publique‑Hôpitaux de Paris (AP-HP), Paris, France; ^4^ Sorbonne Université, INSERM UMR_S 1166, Assistance Publique Hôpitaux de Paris (APHP), Department of Diabetology, Pitié-Salpêtriére Hospital, Institute of Cardiometabolism and Nutrition (ICAN), Paris, France; ^5^ Diabetology – Endocrinology and Metabolism Department, Begin Hospital, Saint-Mande, France; ^6^ Data Science, Withings, Issy-les-Moulineaux, France

**Keywords:** diabetes follow-up, diabetes foot ulcers, electrical skin conductance, sudoscan, withings

## Abstract

**Introduction:**

Diabetic foot ulcers (DFUs) are a major complication of diabetes, leading to high morbidity, mortality, and healthcare costs. Current DFU risk stratification relies on clinical examination, which can be subjective. Electrochemical Skin Conductance (ESC), measured via Sudoscan, offers an objective assessment of small fiber dysfunction. This study evaluates the association between ESC and DFU risk stratification.

**Methods:**

A retrospective analysis of 2,157 diabetic patients from four tertiary centers in France was conducted. DFU risk was classified using the 2016 International Working Group on Diabetic Foot (IWGDF) grading system. ESC measurements were analyzed alongside age, sex, diabetes type, and monofilament test results. Regression and ROC analyses assessed predictive performance.

**Results:**

ESC values correlated with DFU grades (p<0.001), with lower foot ESC (FESC) in higher-risk patients. ROC analysis showed strong predictive value for severe DFUs (AUC = 0.82 for grade 3) but limited performance for early stages. Notably, ESC identified at-risk patients within grade 0, undetected by standard classification.

**Discussion:**

ESC provides a reproducible, operator-independent tool for DFU risk assessment, improving early detection beyond monofilament testing. These findings support its potential role in DFU prevention, reducing amputations and enhancing patient outcomes. Further studies are needed to validate its prognostic value and integration into clinical care.

## Introduction

Diabetic foot ulcers (DFUs) are a pressing global healthcare concern, demanding ongoing attention and innovative approaches to mitigate their devastating impact on individuals with diabetes (1–3). These ulcers represent one of the most distressing complications of diabetes mellitus, predisposing patients to significant morbidity, mortality, and a substantial financial burden on healthcare systems worldwide. Worldwide, there are an estimated 537 million patients with diabetes, and approximately 20% will have any kind of amputation (4). In France, approximately 10,000 amputations per year are due to DFUs, and the cost of diabetes-related treatment is evaluated to be approximately 17.7 billion euros (5, 6).

DFUs are the result of a complex interplay of factors, including neuropathy, ischemia, minor trauma, and other diabetic-related complications. They manifest as chronic wounds on the feet, often resistant to healing, and can lead to infections, limb amputations, and, in severe cases, fatality. The morbidity associated with DFUs is significant, with recurrence rates reaching as high as 65% within 3 to 5 years. Additionally, the lifetime risk of lower-extremity amputations is estimated at 20%, and the 5-year mortality rate is alarmingly high, ranging from 50% to 70%. This escalating health concern is further exacerbated by a troubling increase in overall amputation rates, which have surged by up to 50% in certain regions. This trend disproportionately impacts younger individuals and underserved racial and ethnic minority groups. Despite substantial efforts to improve care for individuals with DFUs, amputation rates have not consistently declined. Moreover, the emergence of disparities in care delivery underscores pressing issues related to equity in diabetes management (2, 7). However, DFUs are not confined to the Western world, where medical healthcare systems are better equipped to record and evaluate diabetic peripheral neuropathy (DPN) due to more robust funding. A recent report by the International Diabetes Federation highlights a growing prevalence of DPN in Africa and South America, paralleling an increase in diabetes cases (8, 9).

In this context, there is a need for an efficient tool to identify subjects at risk of DFU in order to establish early preventive care to avoid DFU manifestation and worsening. The current guidelines for DFU risk stratification are based on clinical examination, but they can be biased by masked symptoms or the ability of clinicians to detect neuropathy and peripheral artery disease. Our study seeks to introduce an innovative approach to DFU risk stratification, with the potential to substantially improve patient outcomes. Our approach is based on the measure of electrochemical skin conductance (ESC) from the Sudoscan device, which has already proven its clinical utility for DPN follow-up (10–16). It provides an objective biological measure for patients and caretakers and is operator-independent and reproducible in current DPN research and guidelines (17).

The aim of this study was to assess the association between ESC measures and DFU risk stratification scores in subjects with diabetes. By providing a detailed account of this innovative approach, we anticipate its potential to enhance the precision of care, improve healing rates, and significantly decrease the incidence of amputations in individuals living with diabetes. Furthermore, our research aimed to contribute to the evolving landscape of DFU management by highlighting the implications for equitable healthcare delivery and patient wellbeing.

## Materials and methods

### Measuring devices

ESC is based on the principle of reverse iontophoresis (18–20) and chronoamperometry. Iontophoresis involves the extraction of ions from the skin; in our case, this process was facilitated by chronoamperometry, which utilizes a stepped electric current. The Sudoscan device includes four large stainless steel plates on which the patients place their hands and feet. These electrodes deliver a low voltage direct current (less than 4 V), which activates the sympathetic innervation of the sweat glands, producing an outflow of chloride ions, which is the origin of a current that will be measured at the level of the electrodes. Thus, the electrodes serve as both stimulation and recording electrodes. The ESC corresponding to this current induced by the chloride ions is expressed in microSiemens (µS) and is objective and quantified data that directly reflects the magnitude and activity of the innervation of the sweat glands by unmyelinated C fibers. The time required for ESC measurement at all four limb extremities takes less than 3 min (21, 22), and no specific training is required to use Sudoscan.

### Dataset

The data from 2,157 patients with diabetes were collected from four different diabetes tertiary centers in France (Bégin, Bichat, Lariboisière, and Pitié) between 2015 and 2019. These centers are part of the Paris University Hospitals (APHP) group. Each of these facilities cares for several thousand patients with type 2 diabetes, 20%–50% of whom have diabetic complications, depending on the type of complication. Data were collected during routine patient follow-ups in their respective diabetes center while performing diabetic daily routine examinations. The examinations include classical blood samples, retinopathy screening, monofilament test, and Sudoscan test for each patient. Patients can also have nutrition and physical activity training and follow-up for specific symptoms or conditions.

### DFU risk stratification system and other variables

In our case, the *risk stratification system* used in the different hospitals was the 2016 version of the International Working Group on the Diabetic Foot (IWGDF) (23):

- grade 0, no sensitive neuropathy;
- grade 1, isolated sensitive neuropathy;
- grade 2, isolated sensitive neuropathy with lower limb arteriopathy and/or foot deformities; and
- grade 3, previous foot ulcer (at least 4-week duration) and/or lower limb amputation.

Monofilament tests were conducted following the recommendations of the IWGDF looking for a loss of sensitivity on the three support points. See work from Practical Guidelines on the prevention and management of diabetic foot disease (IWGDF 2019 update) (24). A patient is considered at risk if there is no perception of monofilament on at least one point in two out of three pressure points. Be aware that the latest version available at the moment of the writing is the 2023 updated one (25).

Other variables used in our work included age, biological sex, monofilament test results (0/1), diabetes type, and ESC. ESC can be given at the feet (FESC) or hands (HESC).

### Visualization and statistical analysis

We performed data exploration using R v4.3.2 (26), rstudio build 494 (27), and Quarto v1.2 (https://quarto.org/).

We first built exploratory plots using the ggplot (28), ggpubr (29), and JLutils (30) packages.

Then, we performed regression analysis to check any major confusing epidemiologic parameters. We conducted the regression normality tests using the Anderson–Darling test (31) since we had many samples; we tested model linearity using a Ramsey test (32), residual normality using the Shapiro test, and heteroscedasticity using the Breusch–Pagan test (33).

Finally, we checked ESC classification against DFU grades. We created receiver operating characteristic (ROC) curves using the plotROC package (34), which produces a binary classification based on the one-vs.-all approach. In our case, we used the ESC stratification versus the DFU grade as the gold standard. We computed confidence intervals using the ci function from the pROC (35) library in R.

### Ethical consideration and anonymization

Data were collected during routine medical examinations in the context of diabetic patient follow-up. Thus, the study was not interventional in any case.

Data were anonymized in each center following the General Data Protection Regulation (GDPR) guidelines (36) edited by the French data privacy control institution (37). Randomness was added to ESC and age (0.1 between 0 and 1), and then data were rounded to units, thus following the principle of randomization/generalization.

Then, analysis was conducted individually in each hospital by the referring physician, and the results were aggregated and presented.

## Results

There were 1,261 men and 896 women aged 16 to 92 years. The patients were distributed among four DFU grades: 1,662 persons with grade 0, 347 with grade 1, 95 with grade 2, and 45 with grade 3. Age was well distributed, with 350, 321, 567, 594, and 317 patients in the age groups 39, 40–49, 50–59, 60–69, and 70+, respectively. The detailed data by hospitals and grades are provided in **Tables 1**, **2**. In **Table 2**, a category merging the information on foot electrochemical skin conductance (FESC) and hand electrochemical skin conductance (HESC) and the current −50/50–70/70+ clinical threshold values was added. A patient with FESC and HESC under 50 was categorized low–low, a patient with 35 and 60 was categorized low–average, and so on. The threshold used for our study was 50–70 (lower and upper) as described in the reference paper on the ethnicity effect (38) for Caucasian people.

**Table 1:** The population distribution and structure across the different hospitals from which we collected the data.

Characteristic	Overall, N = 2,149	Bichat, N = 526	Bégin, N = 250	Pitié, N = 370	Lariboisière, N = 1,003
**Age**	55.9 (14.8)	56.8 (13.1)	62.1 (15.9)	55.1 (12.6)	54.2 (15.7)
**HESC**	57.7 (18.2)	57.6 (18.5)	60.6 (16.8)	57.4 (19.2)	57.1 (17.9)
**FESC**	66.3 (17.8)	64.4 (18.3)	64.8 (18.3)	61.9 (19.6)	69.3 (16.1)
Grade					
* Grade 0*	1,662 (77%)	355 (67%)	184 (74%)	264 (71%)	859 (86%)
* Grade 1*	347 (16%)	146 (28%)	38 (15%)	70 (19%)	93 (9.3%)
* Grade 2*	95 (4.4%)	16 (3.0%)	19 (7.6%)	28 (7.5%)	32 (3.2%)
* Grade 3*	45 (2.1%)	9 (1.7%)	9 (3.6%)	8 (2.1%)	19 (1.9%)
Sex					
* Male*	1,258 (59%)	308 (59%)	148 (59%)	198 (54%)	604 (60%)
* Female*	891 (41%)	218 (41%)	102 (41%)	172 (46%)	399 (40%)
Monofilament					
* 0*	1,842 (85%)	445 (84%)	184 (74%)	322 (87%)	891 (89%)
* 1*	307 (15%)	81 (16%)	66 (26%)	48 (13%)	112 (11%)
Diabetes type					
* Type 1*	550 (26%)	79 (15%)	43 (17%)	136 (36%)	293 (29%)
* Type 2*	1,599 (74%)	447 (85%)	207 (83%)	239 (64%)	710 (71%)
Age category					
39	350 (16%)	62 (12%)	25 (10%)	55 (15%)	208 (21%)
40–49	321 (15%)	82 (16%)	25 (10%)	66 (18%)	148 (15%)
50–59	567 (26%)	156 (30%)	49 (20%)	109 (29%)	253 (25%)
60–69	594 (28%)	163 (31%)	71 (28%)	107 (29%)	253 (25%)
70+	317 (15%)	63 (12%)	80 (32%)	33 (8.9%)	141 (14%)

HESC, hand electrochemical skin conductance; FESC, foot electrochemical skin conductance.

**Table 2:** The population repartition and structure across the different grades.

Characteristic	Grade 0, N = 1,662	Grade 1, N = 347	Grade 2, N = 95	Grade 3, N = 45
Age	54.3 (15.3)	60.0 (11.8)	67.3 (10.2)	59.3 (11.0)
HESC	58.8 (17.8)	54.9 (18.6)	54.0 (17.9)	43.5 (19.0)
FESC	68.7 (16.0)	60.2 (19.8)	59.2 (19.6)	39.9 (21.9)
Sex				
* Male*	960 (58%)	210 (61%)	59 (62%)	29 (64%)
* Female*	702 (42%)	137 (39%)	36 (38%)	16 (36%)
Monofilament				
* 0*	1,662 (100%)	137 (39%)	33 (35%)	10 (22%)
* 1*	8 (0.5%)	210 (61%)	62 (65%)	35 (78%)
Diabetes type				
* Type 1*	478 (29%)	48 (14%)	9 (9.5%)	15 (33%)
* Type 2*	11,184 (71%)	299 (86%)	86 (91%)	30 (67%)
Hospital				
* Bichat*	355 (21%)	146 (42%)	16 (17%)	9 (20%)
* Bégin*	184 (11%)	38 (11%)	19 (20%)	9 (20%)
* Pitié*	264 (16%)	70 (20%)	28 (29%)	8 (18%)
* Lariboisière*	859 (51%)	93 (27%)	32 (34%)	19 (42%)
FESC–HESC				
* High–high*	423 (25%)	62 (18%)	12 (13%)	3 (6.7%)
* High–average*	406 (24%)	49 (14%)	17 (18%)	4 (8.9%)
* High–low*	134 (8.1%)	16 (4.6%)	5 (5.3%)	0 (0%)
* Average–high*	83 (5.0%)	18 (5.2%)	6 (6.3%)	0 (0%)
* Average–average*	210 (13%)	59 (17%)	11 (12%)	5 (11%)
* Average–low*	194 (12%)	50 (14%)	13 (14%)	1 (2.2%)
* Low–high*	7 (0.4%)	2 (0.6%)	3 (3.2%)	0 (0%)
* Low–average*	46 (2.8%)	20 (5.8%)	6 (6.3%)	9 (20%)
* Low–low*	161 (9.7%)	71 (20%)	22 (23%)	23 (51%)

We added a category merging the information on foot electrochemical skin conductance (FESC) and hand electrochemical skin conductance (HESC) and the current −50/50–70/70+ clinical threshold values. A patient with FESC and HESC under 50 will be categorized as low–low, a patient with 35 and 60 will be low–average, and so on.

HESC, hand electrochemical skin conductance; FESC, foot electrochemical skin conductance.

**Figure 1:**
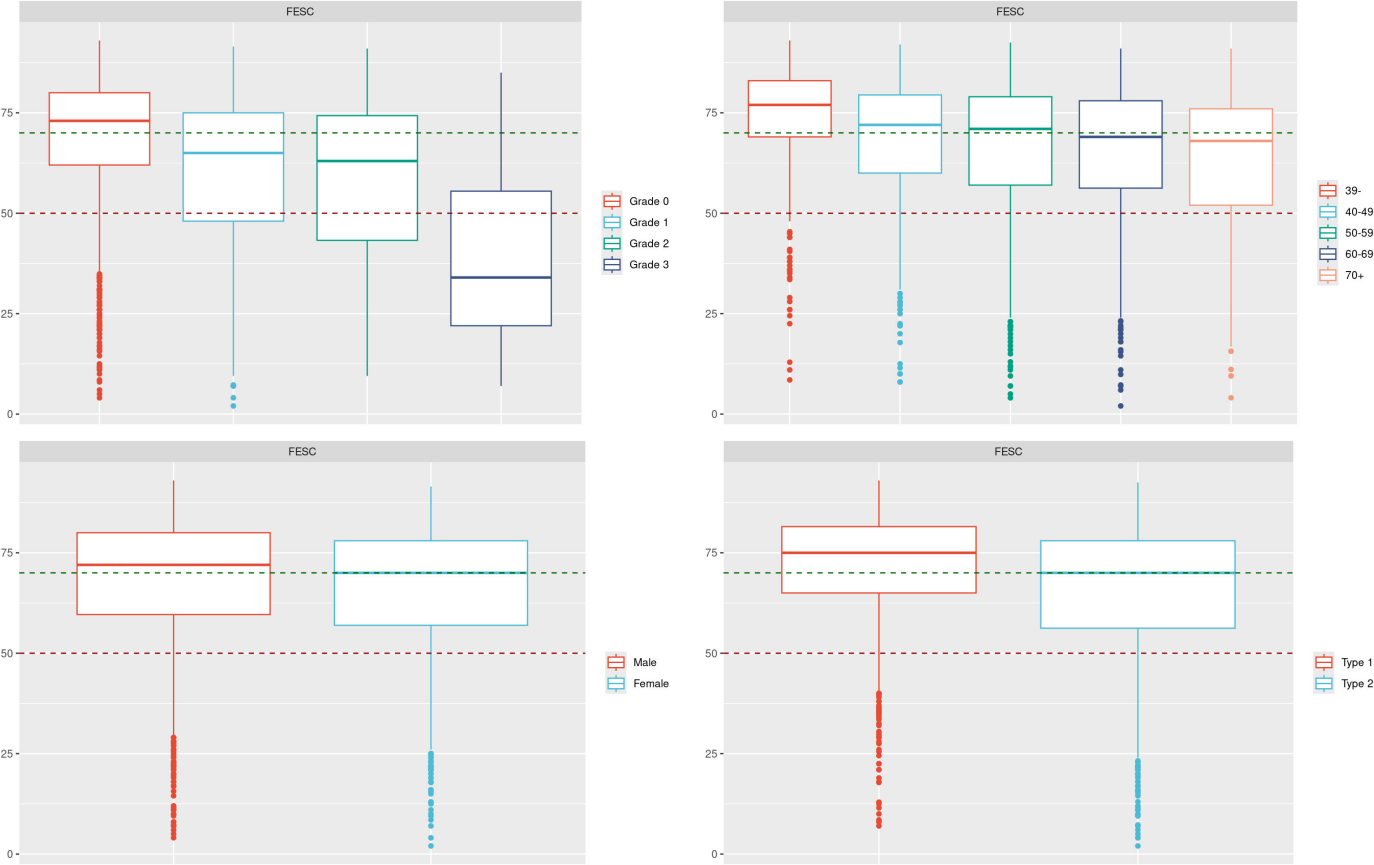
The values of the FESC are split by grade, sex, age category, and diabetes type. The green and red dashed lines represent respectively the known threshold of 70 and 50 for the FESC. FESC, foot electrochemical skin conductance.

Boxplots of the FESC and HESC values were split by grade, age, sex, and diabetes type as presented in **Figure 1**. Associated values can be checked in **Tables 1**, **2**.

The regression model passed all the quality checks, and all results are presented in **Table 3**. Grades were the stronger significant effects, sex was lower (−2.75), and age was even smaller (−0.14). Diabetes type was not significant.

**Table 3:** The results of our multivariate regression analysis performed on our ESC features.

Covariates	ESC	Estimate	Std. error	t value	Pr(>|t|)	Normality	Quadratic association	Residual distribution	Residual homoscedasticity
(Intercept)	FESC	7.8893E+01	1.4639E+00	5.3891E+01	0.0000E+00	3.7000E−24	5.9964E−05	3.1899E−32	1.1329E−08
Age	FESC	−1.4380E−01	2.9578E−02	−4.8619E+00	1.2471E−06	3.7000E−24	5.9964E−05	3.1899E−32	1.1329E−08
Sex, female	FESC	−2.7574E+00	7.3375E−01	−3.7580E+00	1.7586E−04	3.7000E−24	5.9964E−05	3.1899E−32	1.1329E−08
Diabetes type, type 2	FESC	−1.7403E+00	9.9122E−01	−1.7557E+00	7.9281E−02	3.7000E−24	5.9964E−05	3.1899E−32	1.1329E−08
Grade 1	FESC	−7.4976E+00	1.0004E+00	−7.4944E+00	9.6903E−14	3.7000E−24	5.9964E−05	3.1899E−32	1.1329E−08
Grade 2	FESC	−7.4023E+00	1.7962E+00	−4.1211E+00	3.9145E−05	3.7000E−24	5.9964E−05	3.1899E−32	1.1329E−08
Grade 3	FESC	−2.8364E+01	2.5364E+00	−1.1183E+01	2.9528E−28	3.7000E−24	5.9964E−05	3.1899E−32	1.1329E−08

For each feature, we presented the results of the regression per covariate and the control test per regression.

FESC, foot electrochemical skin conductance.

**Figure 2:**
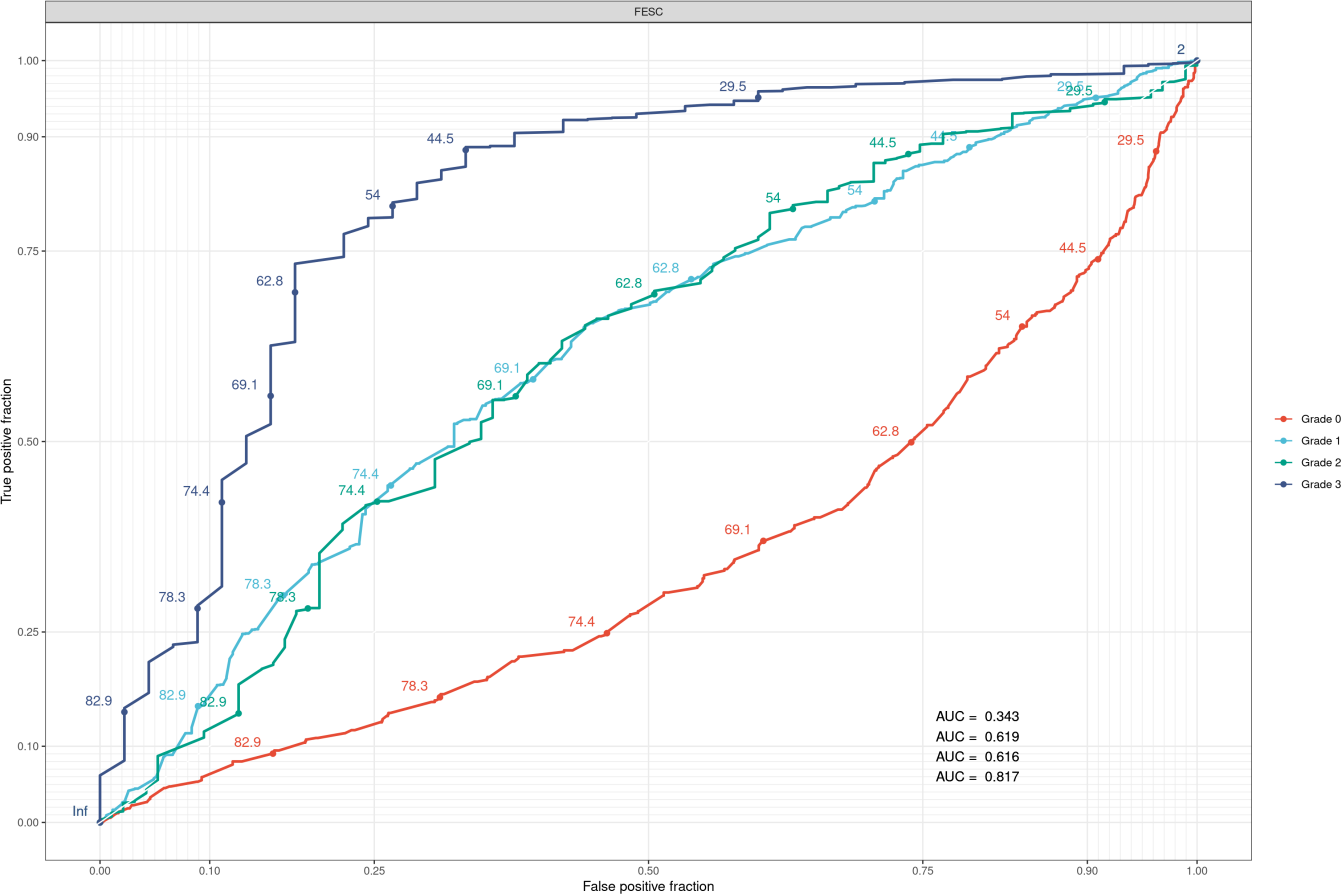
The results of the ROC curve predictions from our FESC feature on the different grades. AUCs on the plots are associated with grades 0, 1, 2, and 3. The AUC for each curve is based on a varying threshold ranging from 0 to 100, trying to classify the data into each grade. The bad AUC curve for grade 0 means that the data are too widespread within the category and that there is no real good threshold to choose from. ROC, receiver operating characteristic; FESC, foot electrochemical skin conductance; AUC, area under the curve.

The area under the curve (AUC) results were 0.82 (95% CI: 0.74–0.89), 0.62 (95% CI: 0.56–0.68), 0.62 (95% CI: 0.59–0.65), and 0.34 (95% CI: 0.35–0.42) for grades 3, 2, 1, and 0, respectively. The results can be checked in **Figure 2**.

## Discussion

In the first intention, we explored graphically our data to see if trends could be observed. In **Figure 1**, we can see the association between the grade and the foot ESC level (FESC) in general. Higher grades have lower values, but we can also notice that grade 0 contains some low values of FESC. FESC values are lower with age, and there is a very small difference between sexes (male/female) with average (SD) FESC values of 67.3 (17.6)/64.9 (17.9). It was expected and already demonstrated in literature as significant but non-clinically relevant (10). In our dataset, we can also observe differences between diabetes type 1 and 2 with FESC values of 70.1 (16.8) and 65.0 (17.9), respectively. Since data were collected during routine examinations, we had no control over the study population and their medical background (medication intake, quality and regularity of medical follow-up, etc.), which means that it is hard to tell whether they were type-specific or population-specific.

Following our exploration, we set up a multivariate analysis to see if the FESC value could be statistically associated with the grade while accounting for the other covariates. The results can be checked in **Table 3**, where we give the details about the model. FESC was quantitatively associated with all the levels of grade, corroborating what we observed graphically. Diabetes type was finally not associated, which means that it was probably confounded with other effects. Sex and age had small significant effects regarding covariate values. The different levels of grade were significant and had the strongest effect on the FESC. Age was an important factor, but it was still confusing because as people age, they are affected by more diseases like chronic kidney disease (CKD) (39, 40) or cardiovascular diseases (CVDs) (41, 42). These conditions (43) can impact ESC values, and in a non-controlled study, we could not dissociate both. These results show a good correlation between our ESC features and the grade system in a global approach corrected for the information we had at our disposal.

We then decided to set up a ROC curve analysis with our FESC feature to see how it could predict the different grades independently of other covariates. The results can be checked in **Figure 2**. As expected for the FESC, we had good predictions for grade 3 and average prediction power for grade 2/1. The predictions for grade 0 were bad because, for the FESC, there was a large range of values. Since the monofilament is not specific (44, 45) during early detection of DFU, we expected these results. Here, the main information was the gradation between the grade predictions that shows a logical association of FESC with worsening DFU states.

**Figure 3:**
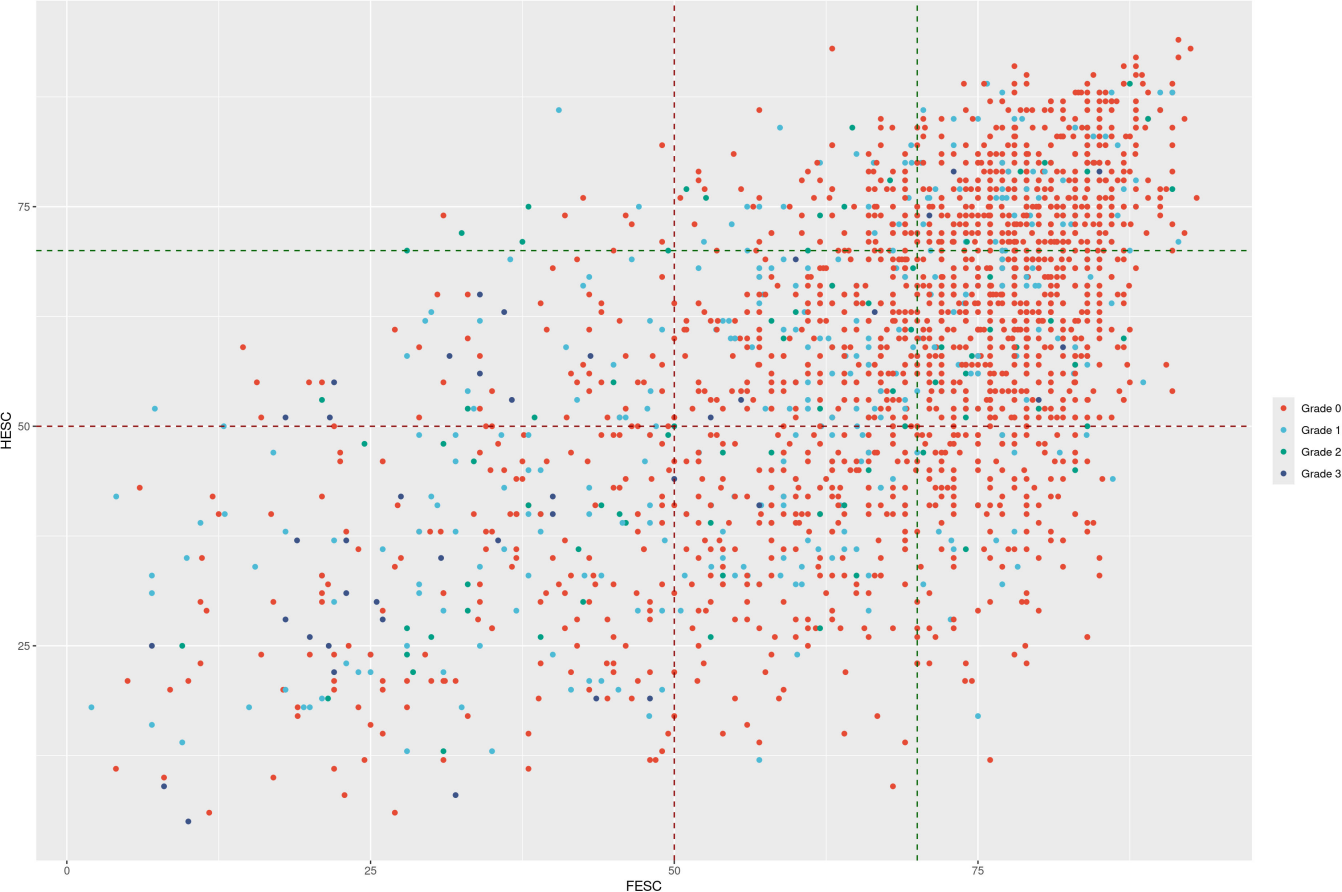
Scatter plot presenting the FESC/HESC on the x/y axis while showing the monofilament test result with point size. The DFU grade is presented in a color scale. With these labels, it is easy to see the patients at risk with low values of FESC or HESC by category while having low-grade and/or negative monofilament tests. FESC, foot electrochemical skin conductance; HESC, hand electrochemical skin conductance; DFU, diabetic foot ulcer.

To show the different granularity caused by the ESC value within the low grade, we present a scatter plot in **Figure 3**. We labeled the data with a built category from FESC and HESC values, which can be a combination of low, average, and high based on their values of 50, 50–70, and 70+, respectively. In this figure, we can see the different groups based on ESC features, with some patients having low FESC and HESC (see also **Table 2**). These patients should be classified as at risk, while with the grading system used, they have a grade 0 classification. These results let us think that grade 0 patients can be clustered into subgroups with more granularity using ESC measures in general.

The recommended risk stratification of DFUs involves intricate evaluations and monofilament tests. However, this test is dependent on the operator’s technique, lacks precision, and is generally poorly reproducible, especially for lower grades of DFU risk (46–48). In this context, ESC may play a pivotal role in predicting the risk of DFUs. Recent studies have already demonstrated its prognostic value (49) against DFU, and it has already been correlated to intraepidermal nerve fiber density (IENFD) (50, 51) while being the subject of positive recent meta-analysis (52).

Our research has shown a quantitative association between ESC features and the high DFU grades but also revealed that ESC offers better granularity in the lower grades of DFUs (grade 0), allowing for improved disease anticipation. This increased granularity is expected, as the technique assesses the health of small C fibers, which deteriorate before larger sensory fibers are detected by the monofilament test. It is important to note that with ESC as a diagnostic measure, 13% of grade 0 patients have already developed neuropathy, and 30% are advised to remain under active follow-up (**Table 2**). This means that ESC has the potential to be a valuable tool for anticipating and preventing the manifestation of DFUs while lowering the economic burden associated with high DFU grades and amputations. It could be also beneficial for remote patient monitoring, thanks to recent advancements in scale technology integration (53) if the care pathway is well coordinated.

## Conclusion

In conclusion, the prevalence and impact of DFUs underscore the urgency for innovative approaches to their prevention. This study unveils ESC measurements as a promising tool for refining DFU stratification risk, exhibiting associations with established risk stratification systems in advanced cases. ESC’s capacity to propose more granularity during the early stages signifies its potential to profoundly influence patient outcomes by potentially curbing amputation rates and advancing proactive intervention strategies. By offering a more precise and objective assessment, ESC holds promise in transforming the landscape of DFU care, paving the way for more tailored and effective interventions. This research illuminates ESC’s pivotal role in redefining DFU management, ultimately contributing to improved healthcare delivery and better quality of life for individuals navigating the complexities of diabetes-related complications. However, while ESC has already been shown to be effective in detecting DPN (54, 55), to the best of our knowledge, as of 2024, only one study has evaluated its role in assessing long-term DFU and mortality risk (56). More research is needed in this area, as it would facilitate the inclusion of ESC in medical-economic studies, allowing it to be integrated effectively into patient care and follow-up.

## Limitations

As with all retrospective observational studies, we are limited by the bias over which we have no control and the lack of confounding medical factors at our disposal. Even if our population was generally well-balanced, all conclusions should be taken according to this type of study. The major problem associated with this work is that we do not have the ethnic information, which is important when measuring electrochemical skin conductance (different thresholds). Age-related diseases can also impact ESC values in a negative way, and we considered here that the main source of low ESC values was DPN since we are working with a known diabetes population.

## Data Availability

The data analyzed in this study is subject to the following licenses/restrictions: Data could be shared upon reasonable request. Requests to access these datasets should be directed to the primary author.

## In memoriam

We would like to thank the late Prof. Ronan Roussel, who initially led this project. We share our most sincere regrets with his family and friends while thanking him for the work he has conducted for his patients with diabetes and medical research.
